# Ultrasound-Determined Residual Gastric Volume after Clear-Fluid Ingestion in the Paediatric Population: Still a Debatable Issue

**DOI:** 10.3390/children9050639

**Published:** 2022-04-29

**Authors:** Mohd Zaid Abdul Kadir, Saw-Kian Cheah, Aliza Mohamad Yusof, Faizah Mohd Zaki, Rufinah Teo

**Affiliations:** 1 Department of Anaesthesiology and Intensive Care, Universiti Kebangsaan Malaysia Medical Centre, Jalan Yaacob Latif, Bandar Tun Razak, Cheras, Kuala Lumpur 56000, Malaysia; mozaic12@yahoo.com (M.Z.A.K.); skii_cheah@yahoo.com (S.-K.C.); alizamyusof@gmail.com (A.M.Y.); 2 Department of Radiology, Universiti Kebangsaan Malaysia Medical Centre, Jalan Yaacob Latif, Bandar Tun Razak, Cheras, Kuala Lumpur 56000, Malaysia; drfaizah@ppukm.ukm.edu.my

**Keywords:** ultrasonography, fasting, gastric antrum, pulmonary aspiration

## Abstract

Background: Current fasting guidelines are often exceeded in clinical practice, resulting in stressful events during anaesthesia in children. This prospective study compares residual gastric volume after 1 versus 2 h of clear fluid ingestion in fasted children. METHODS: A total of 106 patients were enrolled in the study. Ultrasonography (USG) of gastric antrum (GA) was performed in the supine and right lateral decubitus (RLD) positions. All children fasted from solid food for 6 h. Blackcurrant flavoured drink (3 mL/kg) was given following the measurement of baseline (T_0_) USG of GA, with follow-ups after 1 (T_1_) and 2 (T_2_) hours post-ingestion. Residual gastric volume (RGV) was calculated from the cross-sectional area of GA using a standard formula. Parental satisfaction with their children’s behaviour concerning fasting time was recorded. Results: RGV was significantly higher at T_1_ compared to T_2_ (*p* < 0.001). No significant difference was seen between T_0_ and T_2_ (*p* = 0.30). Parental satisfaction was similar at T_1_ and T_2_ (*p* = 0.158). Conclusions: The RGV in paediatric patients after 1 h of clear fluid ingestion was significantly higher than after 2 h of ingestion. There was no difference observed in parental satisfaction concerning the two intervals of fluid fasting. RLD and supine positions can be used reliably to measure the RGV in children.

## 1. Introduction

Aspiration of gastric contents is a potentially devastating cause of morbidity and mortality in all age groups during general anaesthesia. The overall incidence of pulmonary aspiration in children and adults during general anaesthesia has not changed over the last few decades, quoted to be between 0.1% and 19%, depending on the patient and surgical factors [1,2,3]. A recent multicentre survey over a one-year period in specialist paediatric hospitals in the United Kingdom revealed a low incidence of pulmonary aspiration, at 2 per 10,000 in elective cases and 2.2 per 10,000 in emergency cases [4]. One of the risk factors for pulmonary aspiration during general anaesthesia is the volume of the aspirated gastric contents, which leads to pulmonary damage. A study has reported that the presence of a gastric fluid volume greater than 0.8 mL/kg before induction of anaesthesia could lead to the symptomatic pulmonary aspiration of gastric contents due to regurgitation [5]. Therefore, adequate preoperative fasting is one of the important measures to reduce the risk of aspiration during general anaesthesia.

Current paediatric guidelines for preoperative fasting recommend up to 2 h for clear fluids, 4 h for breast milk as well as 6 h for formula milk and solid food [6,7]. Despite these well-established guidelines, a recent study reported by Buller et al. showed most children are fasted for more than the intended period and are fasted for much longer than required [8]. Prolonged fasting time in children increases the risk of preoperative dehydration and hypoglycaemia. A recent consensus statement on the consumption of clear fluids by elective paediatric patients recommended that all children ingest clear fluids up to 1 h before undergoing general anaesthesia [9]. By reducing the fasting time for clear fluids closer to surgery, children experience less thirst, hunger, and anxiety, which translates into a more comfortable and better-behaved child [10]. In addition, overall parental satisfaction also improved by shortening the fluid fasting time. However, there are limited studies to indicate whether 1 h after clear fluid ingestion changes residual gastric volume (RGV) significantly as compared to that of 2 h fasted children.

Several methods that are mentioned in the literature can be used to assess the RGV. Invasive procedures such as manual gastric aspiration via an orogastric tube or endoscopic suctioning in different positions have been advocated [11]. Additionally, imaging studies, such as ultrasonography (USG) and magnetic resonance imaging, can also predict RGV reliably. Although manual gastric aspiration measures RGV more accurately and has a higher sensitivity, it has a higher risk of complications, such as iatrogenic injury to the patient and can be difficult to perform, especially in conscious children [12]. Compared with the manual aspiration of gastric content, gastric ultrasound is a point of care tool that evaluates gastric content at the bedside, both qualitatively and quantitatively [13]. Several studies have concluded that this method can accurately predict the RGV, being highly reproducible with high intra- and inter-rater reliability [14,15,16]. Furthermore, a recent study reported that there was a significant positive relationship between aspirated fluid volume and the cross-sectional area of the gastric antrum, as measured using a USG in determining the RGV [12]. Therefore, the gastric USG can be an important help for anaesthesiologists to minimise the risk of pulmonary aspiration preoperatively, especially in a patient with a high risk of aspiration.

We performed this prospective study primarily to evaluate the RGV after 1 and 2 h of clear fluid fasting. The secondary outcome of this study was to evaluate parents’ satisfaction concerning clear fluid fasting time at 1 and 2 h.

## 2. Materials and Methods

This prospective and single-operator study was conducted during the period from May 2019 to February 2020, in the paediatric wards of a tertiary university hospital, following approval from the institutional research committee of the medical research and ethics committee (JEP-2019-171). All non-surgical paediatric patients who were recruited, admitted, and fasted for various reasons were between 1 and 12 years old, having an American Society of Anesthesiologists (ASA) physical classification system status of I or II, and with body mass indexes (BMI) of less than 30 kg/m^2^. Patients with any known gastrointestinal disorders were excluded from the study.

Following written informed consent from the parents or guardians, patients were instructed to fast for 6 h for solids and non-clear fluids, 4 h for breast milk, and 2 h for clear fluids. A sweetened blackcurrant-flavoured drink was offered as a clear fluid prior to the USG examination. The amount of drink was given under parental supervision, according to their respective age, following our local institutional protocol. For children 1 to 5 years old, they were given from 3 mL/kg up to 55 mL, for those 6 to 12 years of age, up to 140 mL, and for those older than 12 years, up to 250 mL was given.

**Figure 1:**
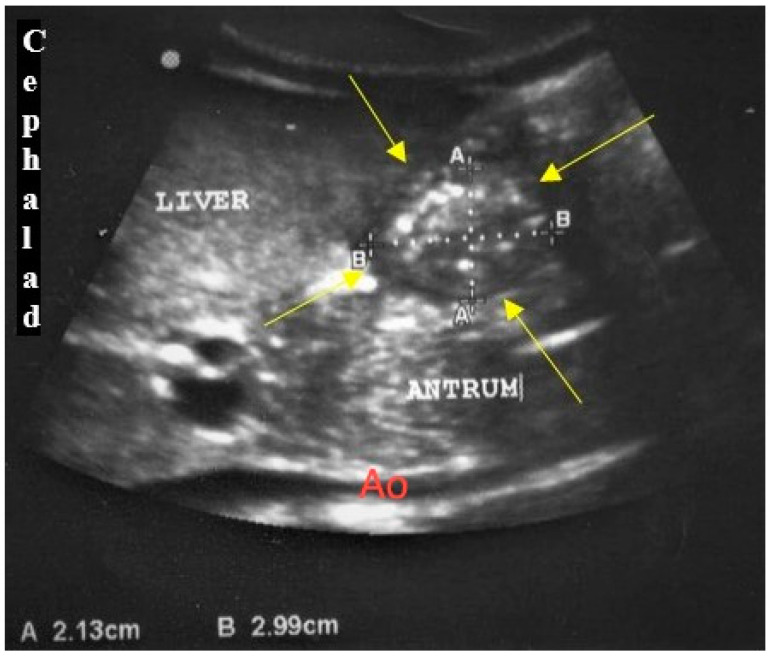
Gastric antrum filled with fluid; A: anteroposterior dimension; Ao: aorta; B: craniocaudal dimension.

The USG examination was performed by a single operator who had undergone USG training under the supervision of a paediatric radiologist. A previously described standardised USG protocol was followed. A two-dimensional USG assessment was performed using a portable ultrasound (Mindray DCC-40) with either a low frequency (2–5 MHz) curvilinear array transducer or a high frequency (8–13 MHz) linear array transducer, as per age and body size recommendation [12]. All patients were first scanned in the supine position, followed by the right lateral decubitus (RLD) position. The gastric antrum was identified in the sagittal to the right parasagittal plane between the left lobe of the liver and the pancreas, at the level of the aorta and superior mesenteric artery or inferior vena cava. The antrum appeared round to oval in shape resembling a bull’s eye target, in which the cross-sectional area (CSA) was measured (Figure 1). Following that, the image of the antrum in an axial plane was obtained to calculate the distance between the anterior and posterior walls. All images were obtained between peristaltic contractions. The RGV was calculated based on the sagittal plane, measuring the craniocaudal (CC) dimension in centimetres (cm) and width (W) in cm, while an axial plane was used to obtain the depth or anteroposterior (AP) dimension, in cm. Thus, the measurable RGV, in millilitres (mL), was calculated using a standard formula; volume (mL) = AP × W × CC × 0.5 (0.5 was the constant number, *k,* considering the shape of the cavity was ellipsoidal) [17].

Each patient underwent USG on three different occasions. First, a baseline USG measurement of gastric antrum (T_0_) was performed before clear fluid ingestion. Subsequent measurements were done 1 h (T_1_) and 2 h (T_2_) post-ingestion. Parental feedback concerning their children’s behaviour during the fasting time was given out after completion of the USG examination. An evaluation was done using a 4-point Likert scale, ranging from 0 to 3 [10]. Scale point 0 was defined as “not at all satisfied”, scale point 1 was “less satisfied”, scale point 2 was “satisfied”, and scale 3 was “very satisfied”. Children who were uncooperative during the USG examination and refused to drink the prescribed fluid according to the protocol or any violation of the fasting guideline times were considered as dropouts.

### Statistical Analysis

The sample size was calculated based on a previous randomised-controlled trial by comparing two paired means of RGV after 1 versus 2 h of clear fluid ingestion in fasted children [10]. Power calculations suggested a sample size of at least 106, including a 10% dropout rate, to detect a difference in RGV, using Student’s paired *t* test at a two-sided significance level of 0.05 and a power of 90%.

All data analysis was performed using SPSS for Windows version 25.0 (IBM Corp, Armonk, NY, USA). Descriptive statistics were used for continuous data. The assumption of a normal distribution of continuous variables was checked with the Shapiro-Wilk test. If variables were normally distributed, the central tendency was expressed as mean and standard deviation (SD). If the variables were not normally distributed, median and inter-quartile ranges were used. Categorical data were expressed as count and percentages or ratios, as appropriate. The Student’s paired *t* test or Wilcoxon signed-rank test was used for normally distributed continuous data and for not normally distributed data, respectively, to test differences in RGV and parental feedback. The Pearson’s correlation analysis was used to evaluate correlations among age, weight, height and the volume of clear fluid drunk with the RGV. The Bland-Altman plot analysed the agreement between RGV taken in the supine and RLD positions. A *p*-value < 0.05 was considered statistically significant.

## 3. Results

A total of 106 patients were enrolled in this study. Of these, 7 children had to be excluded from analysis for the following reasons; violation of fasting time (6 children) and amount of clear fluid allowed exceeded by more than 50% (1 child).

Demographic data of the subjects, baseline gastric volume and volume of ingested fluid are summarized in Table 1 and were normally distributed.

The gastric antrum was visualised in all patients in both supine and RLD positions. The Student paired *t*-test analysis demonstrated the mean and standard deviation of RGV were significantly different between 1 and 2 h after clear fluid ingestion (*p* < 0.001), as shown in Table 2. Similarly, the mean and standard deviation of RGV were significantly different between baseline and 1 h after clear fluid ingestion (*p* < 0.001). However, when 2 h after clear fluid ingestion and baseline were compared, there was no significant difference found in the RGV of the patients in both the supine (*p* = 0.30) and RLD positions (*p* = 0.05).

**Figure 2:**
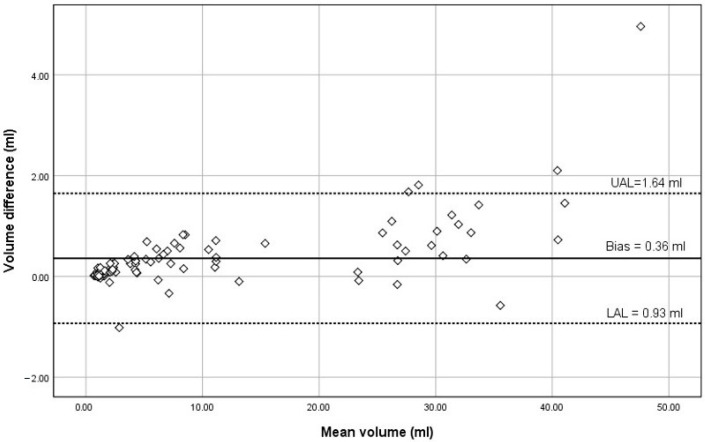
Bland-Altman analysis of RGV taken in supine and RLD positions. The *y*-axis represents the difference between RGV in supine and RLD. The *x*-axis represents the mean between RGV in supine and RLD. The dashed lines are the upper (UAL) and lower limits (LAL) of a 95% agreement band (mean ± 2 SD). The solid line represents the mean difference or bias of the RGV.

The RGV at T_0_ and T_1_ have strong positive correlations with patient age (*r* = 0.810 and *r* = 0.805, respectively, *p* < 0.001) and weight (*r* = 0.853 and *r* = 0.840, respectively, *p* < 0.001). Patient height also positively correlated with RGV at T_0_ and T_1_ (*r* = 0.736 and *r* = 0.760, respectively, *p* < 0.001). However, there was no correlation between the amount of clear fluid ingested and RGV at T_1_ and T_2_. As depicted in Figure 2, the Bland-Altman analysis was performed between the RGV taken in both positions [18]. The mean value of RGV taken in RLD was 0.36 mL higher than that in the supine position and not clinically significant. Thus, the RGV taken in both positions were in agreement with each other.

There was no significant difference between parental satisfaction during T_1_ and T_2,_ as shown in Table 3.

## 4. Discussion

The main finding of our study was that the ingestion of clear fluid changed the RGV significantly when comparing T_1_ and T_2_. The gastric antrum can be consistently identified using the USG with the measurement taken in supine and RLD positions. The parental satisfaction with their child’s behaviour was similar concerning the clear fluid fasting times of 1 h and 2 h.

It is highly desirable and clinically relevant to achieve safe RGV after clear fluid ingestion preoperatively to prevent adverse effects, especially pulmonary aspiration. In recent years, a consensus on perioperative fasting for elective surgery in paediatric patients recommended 1 h of clear fluid fasting before undergoing any procedure requiring general anaesthesia, unless clinically contraindicated [9]. Although the consensus recommended for shorter clear fluid fasting times, based on a study reported by Schmidt et al., data from a larger paediatric population are still required to provide the evidence with respect to safety and comfort aspects [10]. In contrast, our study revealed a significant difference between 1 and 2 h post clear-fluid ingestion in fasted children. A previous study demonstrated that clear fluid ingestion until 2 h before surgery was safe [19]. This report was consistent with our findings and in accordance with the current fasting guidelines, which recommended oral intake of clear fluid up to 2 h prior to elective procedures as it does not increase the gastric fluid content [13]. This study’s findings also underlined the impact of ingested fluid volume on RGV, which has been used as a determinant for the risk of pulmonary aspiration during induction of anaesthesia. The risk volumes were defined previously but other factors may be relevant [12], and the value of the RGV determination has been questioned [20]. A recent multicentre observational study concluded that although the incidence of pulmonary aspiration and regurgitation was not affected by the shortened period of clear fluid fasting, younger patients and those with an emergent status seem to be otherwise [21]. In the most recent pre-operative fasting guidelines from the European Society of Anaesthesiology and Intensive Care (ESAIC), although healthy children are encouraged to drink up to 1 h prior to anaesthesia for elective procedures, there were no randomised, controlled trials assessing the risk of pulmonary aspiration [22]. Therefore, this study is timely and may serve as an adjunct to the existing data that is available.

To date, the clinical application of USG to assess gastric volume has not commonly been practised in clinical settings. The use of ultrasound for the measurement of gastric volume is considered to be safe and the technique described here was non-invasive and readily repeatable at the bedside [15]. Direct RGV measurement can be made at frequent intervals after a drink. In our study, the gastric antrum was consistently identified in both supine and RLD positions by USG in all patients, as per previously obtained and published results [13,23]. Our data demonstrated that mean RGV from USG measurement were within the range of previously observed values, as summarised by Brady et al. [24]. Furthermore, our results also showed the USG measurement of RGV in supine positions was comparable to the RLD position, which was supported by the previous studies as reported in a systemic review by Van de Putte et al. [14]. As such, USG can be used as a point-of-care tool to assess aspiration risk, particularly in situations when fasting status is unknown or uncertain.

There have been few studies in the literature discussing the methods of residual gastric volume on ultrasound prior to the commencement of our study, which then continued to be discussed in the most recent publication by Kim et al. [25] and Tan et al. [26] in 2021 and 2022, respectively. Gastric volume is more representative by measurement using a standard formula, as the stomach is a 3-dimensional cavity and thus the formula, also known as prolate LWH (length X width X height) formula, has been widely used in clinical practice. We did not report on the CSA of the antrum as it only represents a 2-dimensional rather than 3-dimensional measurement. Nevertheless, a new formula has been proposed in a study by Kim et al., published in 2021, which predicted volume (mL) = −3.7 + 6.5 × (RLD CSA [cm^2^]) − 3.9 (supine CSA [cm^2^]) + 1.7 × grade, at the time when our study was completed (Kim et al.). We advocate a standard formula be established in the near future so that this ultrasound practice can be implemented in clinical practice.

Our study showed that RGV exhibited strong positive correlations with age, weight, and height. On the other hand, there was no correlation between the amount of clear fluid intake and RGV during fasting and 1 h and 2 h post-ingestion, which were similarly reported by Song et al. [17]. As our study also found the measurement difference in RGV taken in the RLD and supine positions had no clinical significance [18], either position could be used, as suggested in the systemic review by Van de Putte et al. [14].

We did not find any significance concerning parental satisfaction with their children’s behaviour when compared between 1 and 2 h of clear fluid fasting. As the interval differed by only 1 h, thirst may not have had much impact on the child’s behaviour, as suggested in a study conducted by Schmidt et al. [10]. Furthermore, the clear fluid that was offered was a sweetened blackcurrant-flavoured drink; its caloric content resulted in delayed gastric emptying. This is consistent with a study conducted by Du et al. [27].

There are a few points that need to be emphasised in this study. Firstly, the method of gastric volume calculation was performed on non-surgical patients, assuming normal gastric anatomy and a BMI of up to 30 kg/m^2^, and as such should not be standardised for the general paediatric population. The formula used in this study also should not be generalised to other patients who exhibit underlying medical problems. Further investigations are required in order to standardise the formula for a wider population. The usage of USG on the gastric antrum is limited by the dynamic nature of the organ in which peristaltic contraction and gastric emptying continuously occur after clear fluid ingestion. This may lead to variability between successive measurements, indirectly lowering the reliability of the result.

## 5. Conclusions

The residual gastric volume in paediatric patients after 1 h of clear fluid ingestion is significantly higher when compared to 2 h post-ingestion. This finding could serve as a preliminary guide towards a larger and specific cohort of the population before more liberal fasting guidelines are accepted widely.

## Figures and Tables

**Table 1:** Demographic characteristics of the study population.

Parameter	Mean ± SD	N (%)
Age (years)	5.8 ± 1.81	
Gender		
Male		64 (64.6)
Female		35 (35.4)
* ASA Class		
I		71 (71.7)
II		28 (28.3)
Height (m)	1.1 ± 0.12	
Weight (kg)	20.9 ± 4.61	
Department		
Medical		42 (42.4)
Surgical		57 (57.6)
Volume of fluid ingested (mL/kg)	3.60 ± 0.40	
Baseline gastric volume by weight (mL/kg)	0.19 ± 0.22	

Data are expressed as mean ± SD or number (%) of subjects. * ASA: American Society of Anesthesiologists Classification.

**Table 2:** Mean RGV taken in supine and RLD.

Positions	T_0_ (N = 99)	T_1_ (N = 99)	T_2_ (N = 99)	*p*-Value		
				T_0_ vs. T_1_	T_0_ vs. T_2_	T_1_ vs. T_2_
Supine (mL)	4.75 ± 6.43	10.05 ± 12.19	4.78 ± 6.46	<0.001	0.300	<0.001
RLD (mL)	4.83 ± 6.55	10.41 ± 12.61	4.93 ± 6.58	<0.001	0.05	<0.001

Data are expressed as mean ± SD or (number) of subjects. *p* < 0.05 was accepted for statistically significant.

**Table 3:** Parental satisfaction scale concerning clear fluid fasting time.

Scale	T1, N (%)	T2, N (%)
0	0 (0)	0 (0)
1	4 (4.0)	3 (3.0)
2	46 (46.5)	46 (46.5)
3	49 (49.5)	50 (50.5)

Data are expressed as the number (%) of subjects. *p* = 0.158 compared between T_1_ and T_2_ using Student’s paired *t* test.

## Data Availability

The data presented in this study are available on request from the corresponding author.
